# Impact of Resident-Paired Schedule on Medical Student Education and Impression of Residency Programs

**DOI:** 10.5811/westjem.2020.12.48761

**Published:** 2020-12-19

**Authors:** Ibrahim Mansour, Sean Dyer, Neeraj Chhabra

**Affiliations:** Cook County Health and Hospital Systems, Department of Emergency Medicine, Chicago, Illinois

## Abstract

**Introduction:**

Clinical rotations in emergency medicine (EM) can be challenging for medical students because of the lack of continuity with attending physicians. To overcome this challenge, institutions have started to match a student’s schedule with that of a resident, referred to as “paired shifts.” We sought to pilot and compare two schedule formats for fourth-year medical students (MS4) – a resident-paired shifts (RPS) and a traditional resident-unpaired shifts (RUS) schedule.

**Methods:**

This prospective, crossover trial included MS4s rotating in the emergency department over four consecutive four-week blocks. Each MS4 was assigned two weeks using the RUS schedule and two weeks with the RPS schedule, alternating the format order each month. At the end of the rotation students were anonymously surveyed regarding the differences in learning experience, their ability to showcase their knowledge and clinical skills, and familiarity with the residency program with the two formats.

**Results:**

The response rate was 47 of 58 students (84%). Respondents indicated that RPS resulted in more teaching time (64.6% RPS vs 8.3% RUS), a better overall educational experience (68.8% RPS vs 8.3% RUS), and a greater ability to showcase their medical knowledge (52.1% RPS vs 6.3% RUS). Additionally, students felt that the program was better able to evaluate them (66.7% RPS vs 10.4% RUS) and they were better able to better evaluate the program (66.7% RPS vs 6.3% RUS) in the RPS format.

**Conclusions:**

When compared to traditional RUS during an MS4 rotation, a RPS format provided students with the perception of an improved learning experience, ability to showcase knowledge, and familiarity with the residency program without sacrificing teaching from attending physicians.

## INTRODUCTION

Integrating medical students into a busy emergency department (ED) is often challenging.^1^ While the ED offers hands-on learning experiences for students, some of these opportunities may be lost due to the pace of the ED and clinical demands of the providers. Student exposure to emergency medicine (EM) is limited in the preclinical years, and a clinical rotation provides an opportunity for students to explore a potential career in EM.^2^,^3^ For students who have decided or may decide to apply for residency training in EM, these clinical rotations in the fourth year of medical school, commonly known as “audition” rotations, are available at the majority of EM residency programs. These rotations give applicants the opportunity to compare and contrast different EM residency programs, obtain a Standard Letter of Evaluation (SLOE), and to “audition” for a spot in the residency program by highlighting their individual characteristics.^4^

One obstacle to medical students’ educational and potential “audition” experience in EDs is the frequent changes in supervising emergency physicians due to shift scheduling. Students typically work with multiple resident and attending physicians, but might not work with the same physician more than once. While this allows medical students to interact with more emergency physicians, this lack of continuity may provide little opportunity for medical students to highlight personal growth and implementation of feedback. These difficulties can be compounded in busier EDs such as ours with 68 residents and over 135,000 visits annually. It is also unclear to what degree this lack of continuity with supervising physicians affects on-shift teaching, learning content, and overall impressions of residency programs.

Medical student clinical rotations in the ED are traditionally formatted such that students are assigned shifts in the department irrespective of the schedules of supervising physicians, either resident or attending. To increase continuity between students and supervising physicians, some programs have begun to match students’ schedules with those of specific resident physicians.^5^ Bernard et al found that a small cohort of students rated frequency and quality of feedback, interactions, and teaching superior with their “continuity-based shift model,” similar to our RPS format.^5^ Historically, our program has employed an unpaired scheduling format. In the current study we sought to compare a traditional, unpaired medical student/resident schedule with a paired schedule. Specifically, we compared medical student perceptions of the two schedule formats in regard to learning experience, “audition” opportunity, and familiarity gained with the residency program.

## METHODS

This was a prospective crossover trial at an Accreditation Council for Graduate Medical Education (ACGME)-accredited postgraduate year (EM, PGY1-4) residency program from May–August, 2019. The purpose was to evaluate two different rotation schedule designs for fourth-year medical students (MS4). The hospital system does not have an affiliated medical school, and MS4s from multiple medical schools rotate in the ED. We chose this time frame because it represents a common time period in which MS4s applying to EM residency programs for the following year (2020) would complete their “audition” rotations.^2^

MS4s rotating in EM over four consecutive four-week blocks were assigned two weeks using a traditional block schedule with resident-unpaired shifts (RUS) and two weeks with resident-paired shifts (RPS). For two of the four blocks, the MS4s rotated first using the RUS schedule for two weeks followed by the RPS schedule for two weeks. For the other two blocks, this order was reversed in order to diminish the potential bias based on the order of the two schedule types.

The RUS schedule involved MS4s choosing a predesigned block schedule with changing shift times and locations within the ED without continuity in terms of working with particular residents or attending physicians. The RPS schedule assigned an MS4 with a resident physician in the PGY-3 or PGY-4 year so that they worked the same schedule as the resident physician. The number of shifts worked by each MS4 in both the RUS (seven shifts) and RPS schedules (six shifts) were similar although not evenly matched to allow for all students to have an equal number of total shifts and limitations in department capacity. All shifts in both the RPS and RUS schedule were eight hours in length and under the supervision of an attending physician. To minimize potential bias, all PGY-3 and PGY-4 residents working in the ED during this time period participated in the study if their schedule met the minimum number of required shifts. Resident physicians were advised of the schedule change for students but were given no further instruction.

**Population Health Research Capsule**

What do we already know about this issue?

By increasing continuity between teacher-learner during medical student EM rotations, there is an increase in the amount and quality of feedback a learner receives.

What was the research question?

Do resident paired shifts improve a student’s educational experience, familiarity with the program and audition opportunity?

What was the major finding of the study?

A student’s experience, familiarity with the program and ability to showcase knowledge improve with resident paired shifts.

How does this improve population health?

Resident paired shifts in an EM clerkship improves a student’s perception of their educational experience and ability to learn about a potential residency program.

At the conclusion of the four-week block an anonymous, confidential, and voluntary electronic survey instrument was sent to all participating MS4s. The instrument used closed-ended, ranked Likert-scale responses to evaluate the student learning experience, “audition” opportunity, familiarity with the residency program, and overall preference for schedule type (Appendix A). These domains were specifically chosen due to their importance to both prospective applicants and residency programs. The survey was reviewed by educational faculty with expertise in medical student education and residency recruitment. It was then edited for clarity and relevance to the aforementioned domains of interest based on feedback provided. Additionally, the MS4s were given the opportunity to provide general comments and feedback via a free-response field.

We analyzed the data using SPSS Statistics for Windows, version 26 (IBM Corp., Armonk, NY). For each item, responses were trichotomized and a one-sample two-tailed chi-square analysis tested the null hypothesis of equal preference for the RPS schedule and RUS schedule at a significance level of 0.05. For the purposes of the analyses, responses indicating “more” or “much more” preference for either the RPS or RUS were counted as a single category and compared. In addition to quantitatively analyzing which schedule format respondents preferred, the authors reviewed the free response section for positive and negative comments regarding the two schedule formats and their impact on the aforementioned domains. The study was exempted by the institutional review board.

## RESULTS

Of 57 MS4s 48 completed the survey, representing an 84% response rate. Students indicated more direct teaching time (64.6% RPS vs 8.3% RUS; *P*<0.001) and teaching that was more appropriate for their level of training (50% RPS vs 6.3% RUS; *P*<0.001) with the RPS format, while the amount of teaching time students received directly from the supervising attending physicians was similar in the two groups (14.6% RPS vs 18.8% RUS; *P* = 0.617). In addition, respondents indicated that the resident paired shifts made students more comfortable asking clinical questions (72.9% RPS vs 2.1% RUS; *P*<0.001) and resulted in the perception of a better overall educational experience (68.8% RPS vs 8.3% RUS; *P*<0.001) (Table 1).

In items evaluating the respondents “audition” opportunity, students indicated that they were better able to showcase their medical knowledge (52.1% RPS vs 6.3% RUS; *P*<0.001) and that the program got to know them better as applicants in resident-paired shifts (66.7% RPS vs 10.4% RUS; *P*<0.001) (Table 2). For familiarity with the residency program, the resident-paired shifts led to more opportunities to ask questions about the residency program (56.2% RPS vs 4.2% RUS; *P*<0.001), and students indicated they were better able to evaluate the program in this format (66.7% RPS vs 6.3% RUS; *P*<0.001) (Table 3).

A free-response section allowed students to comment on their experience with the two formats and which they preferred and why. Of the 43 responses to this section, 25 students stated that they preferred the resident format (58.1%), while three stated they preferred the block format (7.0%). Although not presented as an option, 13 respondents (30.2%) stated that they preferred a mix of both formats, especially if they were able to work with a resident for two weeks prior to starting a traditional unpaired block format. Specific comments regarding the advantage of the RPS format included the following: “nice having a familiar face while learning about how the emergency department at Stroger worked”; “gave me the opportunity to ask about the general workflow/thought process in the ED”; and “I really enjoyed being paired up with a resident, but may have appreciated it even more during the first 2 weeks instead of the last 2 weeks.”

Three themes emerged from the free-response section regarding the RPS format: 1) the format allowed for an increased ability to showcase their knowledge; 2) it gave the students the opportunity to demonstrate a progression of their skills over time; and 3) provided them increased familiarity with the residency program. Many respondents indicated that the RPS provided a balance of mentorship/guidance from the resident while still being able to interact with an attending, as represented by the following comment: “I really liked working with one resident because I felt like I was able to still see patients autonomously with an attending but I had one person I knew really well who I could talk to and get advice from.” The few comments favoring the RUS format discussed a more extensive exposure to various teaching styles and viewpoints along with more attending interaction, represented by the following comment: “The block schedule in the beginning exposed me to more residents in the program which gave me a better idea of how I’d fit in, and allowed me to hear from multiple people about their experiences”; “I felt that I missed out on certain learning opportunities towards the second half if someone who was not my assigned senior had a cool case/procedure.”

## DISCUSSION

In this prospective crossover trial, we demonstrate the positive impact of a MS4 schedule format that increased continuity between learner and teacher in the following ways: learning experience; “audition” opportunity; and familiarity with the residency program. The results suggest that a two-week period of RPS fostered a more satisfactory educational experience for the students and “audition” opportunity, as well as perceived familiarity with the residency program.

As the SLOE has become one of the most valued parts of a MS4’s application to prospective residency programs, it is important that a student’s performance is adequately and accurately assessed during an EM rotation.^2^,^6^ This can be difficult given the nature of student scheduling in the ED as a student might work with different faculty and residents each shift, making it hard to demonstrate longitudinal improvement, form relationships, and show the ability to incorporate feedback. Our study demonstrates that two-thirds of rotating students believed the residency program was better able to assess their abilities as a potential applicant and half believed they could better demonstrate their EM knowledge with a resident-paired schedule. However, this study did not examine the residency program’s ability to better evaluate the applicant with either format. The RPS led to 67% of students gaining a better understanding of the residency program, thereby further fulfilling one of the primary objectives of doing an “audition” clinical rotation.^2^ Overall, using the RPS appears to help satisfy the goals of an “audition” rotation better than the RUS.

An essential part of any medical student rotation is to ensure there is appropriate educational content delivered to the student.^3^,^7^ While this can happen in the form of scheduled didactic conferences and independent studying, much of this learning is done during clinical shifts in EM. Compared to the standard RUS, when paired with a resident for part of their rotation the majority of students felt more comfortable asking questions, had more direct teaching appropriate for their level of training, and overall had a better educational experience. By using a RPS schedule, clerkship directors and residency leadership can improve rotating medical students’ satisfaction with their educational experience. Interestingly, direct teaching time from attending physicians was reported by two-thirds of students to be similar with the resident-paired and resident-unpaired schedules. This suggests that despite the perception of an overall increase in direct teaching time with the resident-paired schedule, it was not at the expense of direct teaching from the supervising attending physicians.

While the survey required that students state a preference for either the RPS or the RUS format, the free- response section of the survey allowed for about a third of the respondents to observe that they would favor a combined schedule format, having both he RPS and RUS for two weeks. Originally, having students experience both formats was done for each student to serve as their own control between the two schedule formats, but these responses suggest that the combination of schedules may provide some advantages. From the free responses, it became clear that students preferred having the RPS during the initial portion of a rotation to become better acclimated with a new hospital environment and operations of the ED. Some responses indicated having a familiar resident to whom they could direct questions helped with this process, at least initially. In the second half of the rotation, an unpaired schedule may have helped to expose the students to varying practice and teaching styles. Therefore, a combined schedule format with RPS in the first two weeks and RUS during the last two weeks may be the ideal combination, allowing for the learner to benefit from the advantages of each format and should be further investigated.

## LIMITATIONS

One limitation of this study was the individualized experience each student had with their paired resident as there were at times a disproportionate number of PGY-4 vs PGY-3 residents paired with students, given monthly scheduling limitations. If a certain resident was more adept at teaching on shift or there was a personality mismatch between the student and resident this could have altered the perception and educational experience of the RPS. This study did not examine how these interactions impacted the objective performance or perceptions of the student. Although medical students are aware of being evaluated during all their medical school rotations, there was still the possibility of the Hawthorne effect given that students were made aware of the two formats and the eventual comparison survey during their rotation orientation. While it was mentioned that the resident pairing was new compared to the traditional unpaired shifts and could have led to favoring of this new format, no indication was made as to which format was favored by the clerkship leadership during the orientation.

Another limitation was the subjectivity of each individual’s experience, especially if they were planning on EM as a future career. This could have altered their perception of the quality of the education during the rotation. As this study was conducted at a single institution, the results may not be generalizable to other institutions and should be repeated at other program types and settings to ensure comparable results. Finally, limitations that are inherent in survey studies are likely present in our results. These include interpretation of survey answer choices; lack of memory of the experience while completing the survey the weeks following the rotation’s end; and, the possibility that respondents could have been concerned about commenting negatively about the residency program even though the surveys were anonymous with no answer data identifying a particular student.

## CONCLUSION

We found that medical students perceived a better educational experience and “audition” opportunity, as well as the opportunity to learn more about the residency program with a two-week resident-paired schedule when compared to a traditional resident-unpaired schedule. This type of scheduling should be studied in other settings and program types to ensure comparable results. Medical student clerkship directors might consider including a resident-paired schedule portion in their rotation schedule to improve the students’ satisfaction with their educational experience and provide the preferred format for the student to evaluate the residency program.

## Supplementary Information



## Figures and Tables

**Table 1:** Medical student learning experience comparing resident-paired vs unpaired schedules.

	More or much more with RPS (%)	No difference (%)	More or much more with RUS (%)
Which schedule format allowed you to receive more direct teaching time?*	31/48 (64.6%)	13/48 (27.08%)	4/48 (8.3%)
Which schedule format allowed you to receive teaching that was more appropriate for your level of training?*	24/48 (50.0%)	21/48 (43.75%)	3/48 (6.3%)
Which schedule format allowed for more direct teaching time from attending physicians?	7/48 (14.6%)	32/48 (66.67%)	9/48 (18.8%)
In which schedule format were you more comfortable asking questions about patient care and medical knowledge?*	35/48 (72.9%)	12/48 (25%)	1/48 (2.1%)
Which schedule format allowed you to maximize your educational experience during the rotation?*	33/48 (68.8%)	11/48 (22.92%)	4/48 (8.3%)

*RPS*, resident-paired schedule; *RUS*, resident-unpaired schedule;

* = *P*<0.001.

**Table 2:** Audition opportunity.

	More or much more with RPS (%)	No difference (%)	More or much more with RUS (%)
Which schedule format do you feel allowed the program to get to know you better as an applicant?*	32/48 (66.7%)	11/48 (22.92%)	5/48 (10.4%)
Which schedule format allowed you to demonstrate your knowledge of emergency medicine better?*	25/48 (52.1%)	20/48 (41.67%)	3/48 (6.3%)

*RPS*, resident-paired schedule; *RUS*, resident-unpaired schedule;

* = *P*<0.001.

**Table 3:** Familiarity with residency program.

	More or much more with RPS (%)	No difference (%)	More or much more with RUS (%)
Which schedule format gave you more opportunities to ask questions about the residency program?*	27/48 (56.2%)	19/48 (39.58%)	2/48 (4.2%)
Which schedule format gave you a better ability to learn about and evaluate the residency program?*	32/48 (66.7%)	13/48 (27.08%)	3/48 (6.3%)

*RPS*, resident-paired schedule; *RUS*, resident-unpaired schedule;

* = *P*<0.001.
