# Supplementary material for: Impact of Resident-Paired Schedule on Medical Student Education and Impression of Residency Programs
Source: West J Emerg Med. 2020 Dec 19;22(1):15–9. doi: 10.5811/westjem.2020.12.48761 (PMC7806323; doi:10.5811/westjem.2020.12.48761)
Supplement: Supplementary file 1 [file wjem-22-15-s001.docx]

**Appendix A**

Which schedule format allowed you to receive more direct teaching time?

| Much more with resident schedule | More with resident schedule | No difference | More with block schedule | Much more with block schedule |
| --- | --- | --- | --- | --- |

Which schedule format allowed you to receive teaching that was more appropriate to your level of training?

| Much more with resident schedule | More with resident schedule | No difference | More with block schedule | Much more with block schedule |
| --- | --- | --- | --- | --- |

Which schedule format allowed you to maximize your educational experience during the rotation?

| Much more with resident schedule | More with resident schedule | No difference | More with block schedule | Much more with block schedule |
| --- | --- | --- | --- | --- |

In which schedule format where you more comfortable asking questions about patient care and medical knowledge?

| Much more with resident schedule | More with resident schedule | No difference | More with block schedule | Much more with block schedule |
| --- | --- | --- | --- | --- |

Which scheduling format allowed for more direct teaching time from attending physicians?

| Much more with resident schedule | More with resident schedule | No difference | More with block schedule | Much more with block schedule |
| --- | --- | --- | --- | --- |

Which scheduling format gave you a better ability to learn about and evaluate the residency program?

| Much more with resident schedule | More with resident schedule | No difference | More with block schedule | Much more with block schedule |
| --- | --- | --- | --- | --- |

Which scheduling format gave you more opportunities to ask questions about the residency program?

| Much more with resident schedule | More with resident schedule | No difference | More with block schedule | Much more with block schedule |
| --- | --- | --- | --- | --- |

Which scheduling format allowed you to demonstrate your knowledge of emergency medicine better?

| Much more with resident schedule | More with resident schedule | No difference | More with block schedule | Much more with block schedule |
| --- | --- | --- | --- | --- |

Which scheduling format do you feel allowed the program to get to know you better as an applicant?

| Much more with resident schedule | More with resident schedule | No difference | More with block schedule | Much more with block schedule |
| --- | --- | --- | --- | --- |

For the purposes of education, auditioning, and getting to know the program, which schedule format was better and why?
